# New Compound of Chlorine, Iodine and Mercury, in Scrofula

**Published:** 1846-08

**Authors:** 


*New Compound of Chlorine, Iodine and Mercury, in Scrofula.*—Mr. Rochard has communicated to the Académie des Sciences, Paris, a paper entitled "Trial of a New Compound of Chlorine, Iodine and Mercury, in the treatment of Scrofulous Affections."

The author reports a considerable number of cases, the results of which seem to him to prove that this composition, which M. Boutigni has made known, and designated by the name of "*iodhydrargirite de chlorure mercureux*," exercises an efficient action in scrofulous affections of the most serious character, and also in inveterate cutaneous diseases. He states that after having obtained some rapid cures in psoriasis lichen, chronic eczema, herpes, maculæ, &c., the idea occurred to him of extending its employment to the treatment of scrofula. He cites, amongst others, some successful cases of white swelling, with caries, and fistulous canals; of numerous enlarged indurated or ulcerated ganglia; of chronic ophthalmia, complicated with ulcerating keratitis; of ulcerated lupus; of goitre; and finally of large scrofulous abscesses, succeeding to an anti-syphilitic treatment. In these several cases the action of the remedy was quick and permanent, though varying in the various forms of the disease. M. Rochard employs the medicine externally in the form of ointment.—*Ibid.*

